# Sulfur Mustard Exposure from Dredged Artillery Shell in a Commercial Clammer

**DOI:** 10.5811/cpcem.2017.5.34034

**Published:** 2017-09-29

**Authors:** Jenna Otter, Alveena Dawood, Joseph D’Orazio

**Affiliations:** Temple University Hospital, Department of Emergency Medicine, Philadelphia, Pennsylvania

## Abstract

A 40-year-old commercial fisherman presented with a blistering second degree burn to the right arm after handling a dredged and undetonated World War I-era sulfur mustard artillery shell. He sustained isolated second degree cutaneous injury requiring wound care and skin grafting. Sulfur mustard, or dichlorethylsulphide, is a vesicant chemical warfare agent that causes significant cutaneous chemical burn and is managed with burn wound care. Long-term effects include cosmetic disfigurement and increased risk of developing cancer. Sulfur mustard exposure is a rare but devastating injury when discarded artillery shells are encountered in coastal waters.

## INTRODUCTION

Sulfur mustard, or mustard gas, was a common toxicological exposure among soldiers fighting in World War I, causing severe burns and long-term disfiguration. After World War I, World War II, and until 1970, coastal dumping of unwanted munitions, including shells filled with chemical agents, was commonplace. This practice left an estimated 64 million pounds of nerve and sulfur mustard agents at the bottom of the coastal waters along with countless other chemical agents and undetonated shells.^1^,^2^ Of these agents, sulfur mustard may be the most hazardous in accidental exposure because of its ability to form a solid or semi-solid material that persists for decades.^3^ Sporadic cases of dredged artillery shells along the Atlantic coast have occurred in recent years, making sulfur mustard exposure a patient presentation of which physicians should be aware.

## CASE REPORT

A 40-year-old commercial fisherman with no medical problems was working on a clamming boat 30 miles off the coast when he dredged up a World War I-era artillery shell. He handled the undetonated shell without protective equipment and threw the shell back into the water with his right arm. Within an hour of exposure to the yellow liquid contained in the shell, he developed pain, redness, and blistering from the right hand to the elbow. He immediately sought treatment at the emergency department of a local hospital and was transferred to a burn intensive care unit (ICU).

Upon arrival to the burn ICU, his vital signs were a heart rate of 48 beats per minute, blood pressure of 123/75 mmHg, and oxygen saturation of 98% on room air with a normal respiratory rate. A skin exam revealed 5% total body surface area second-degree partial thickness and first degree burns of the lateral aspect of the right upper arm. His face had no burns, mucous membranes were normal and moist without signs of irritation, lungs were clear to auscultation, and abdominal exam was normal. Laboratory studies were normal other than baseline normocytic anemia.

The patient’s injury was consistent with cutaneous sulfur mustard exposure that had likely leaked out of the old shell he had dredged earlier in the day. He was initially treated with wound debridement and dressing changes with silver sulfadiazine ointment. Split thickness skin grafts to multiple sites over the right arm were performed several days after exposure. The patient was subsequently discharged from the burn service in stable condition with outpatient follow-up.

The incident was reported to the Department of Environmental Protection, which then involved the U.S. Coast Guard, Food and Drug Administration, and Delaware Department of Natural Resources and Environmental Control. The fishing vessel was evaluated but no hazardous materials were found. Since the clams from the vessel had already been delivered to a seafood processing center, over 700 cases of clam chowder were recalled and discarded because of concern for contamination.

## DISCUSSION

Sulfur mustard, or dichlorethylsulphide, dates to before the nineteenth century, when it was used as a pesticide and was explored unsuccessfully for use as a chemotherapeutic agent.^4^,^5^ World War I was the debut of sulfur mustard as an agent of chemical warfare. Exposure was most often cutaneous, causing painful blisters, permanent physical disfigurement, and significant psychological stress. Sulfur mustard was used against civilians in the Iraq-Iran War of the late 1980s.^6^,^7^ It is speculated that U.S. troops were exposed to sulfur mustard in the Gulf War, but no definitive evidence has been found.^8^ Sulfur mustard production in the U.S. ceased in 1968; however, it has since been stored in several sites across the country. Although these sites are guarded and precautions are taken against exposure, multiple incidences of accidental exposure have been reported.^4^

Sulfur mustard is a potent, lipophilic alkylating agent that causes blistering of the skin and mucosal surfaces leading to pain and disfigurement. When used in the world wars, troops were taught to recognize the distinct scent of sulfur mustard and taste of garlic to indicate active exposure. Troops were trained to don gas masks to prevent inhalational injury but cutaneous chemical burns would still occur. Cutaneous exposures most severely affect areas that are rich with eccrine and sebaceous glands on the human body, including the face, axillae and groin, due to its lipophilic nature.^4^,^5^ Upon skin- or mucous-membrane exposure, the chemical is directly absorbed, but injuries do not become apparent until 30 minutes later or may be delayed as late as 48 hours after exposure. Extent of cutaneous injury is dependent on duration of exposure, dose of agent, ambient temperature and humidity of the environment or exposed surface.^4^ Sulfur mustard causes degeneration of the basement membrane, keratinocyte death, and inflammatory changes. Apoptosis ensues and bullae form, filled with yellow fluid.^10^ Initially, melanocytes are stimulated, leading to hyperpigmentation, followed by sloughing of skin and hypopigmentation as skin heals.^4^,^5^,^9^

With more significant exposure or when exposed to its liquid form, sulfur mustard can also affect the respiratory tract, eyes, gastrointestinal tract and hematopoietic system. Inhalation of sulfur mustard causes airway inflammation, epithelial damage and necrosis. These injuries manifest as cough, hoarseness, hemoptysis, excess sputum production, and, in more severe high concentration exposures, pulmonary edema, bronchopneumonia and acute respiratory distress syndrome. Acute manifestations of ocular exposure include acute conjunctivitis, eyelid inflammation, and photophobia. The high metabolic turnover of corneal epithelium causes the eyes to be particularly sensitive to sulfur mustard exposure. Recovery from symptoms due to mild exposure occurs within a few days whereas significant contact may result in temporary blindness. Gastrointestinal exposure has been associated with immediate nausea and vomiting, abdominal pain, and diarrhea. Sequelae of gastrointestinal exposure described include the development of Barrett’s esophagus and some malignancies.^10^ In large cutaneous exposures or systemic exposures, initial leukocytosis followed by leukopenia ensues.^4^

**CPC-EM Capsule**

What do we already know about this clinical entity?

Sulfur mustard, or dichlorethylsulphide, is a vesicant chemical warfare agent that causes significant cutaneous chemical burn and is managed with burn wound care.

What makes this presentation of disease reportable?

Sulfur mustard exposure is a rare but devastating injury that may occur when discarded artillery shells are encountered in coastal waters.

What is the major learning point?

Sulfur mustard may be encountered in clinical practice, and care should be taken in decontamination, appropriate burn care of lesions, and consideration for other injured organ systems.

How might this improve emergency medicine practice?

Emergency physicians should recognize signs of cutaneous sulfur mustard exposure. It may alert them to mass casualties and aid in preventing further harm from secondary exposures.

Sulfur mustard is proposed to exert its effects on the cell through a number of suggested pathways. Its absorption leads to an increase in free radicals and lipid peroxidation, ultimately causing oxidative cellular injury and apoptosis. In one proposed mechanism, sulfur mustard alkylates deoxyribonucleic acid leading to activation of the intracellular repair enzyme poly(adenosine diphosphate-ribose) polymerase (PARP). This exhausts intracellular nicotinic adenine dinucleotide + (NAD+) leading to decreased glycolysis, protease release and cell injury. Upon cell death, proteases are released from the cell, causing dermal-epidermal separation as blisters. In another proposed mechanism, sulfur mustard inactivates glutathione, which leaves the cell vulnerable to oxidative injury and leads to increased intracellular accumulation of calcium and ultimately cell death.^4^ The etiology of acute and delayed toxicity of sulfur mustard exposure is not fully elucidated.

The emergency physician may suspect an isolated cutaneous sulfur mustard burn after obtaining a detailed history of exposure, but in the absence of this information the yellow fluid contained in the bullae and history of delayed development of burn after exposure to a chemical may act as clues to diagnosis. In a mass casualty scenario, reports of garlic or onion smell at the scene with delayed presentation of mucocutaneous injuries with the characteristic yellow fluid-filled bullae may alert the clinician to sulfur mustard exposure. When exposure to sulfur mustard is suspected, immediate decontamination including removal of clothing and scrubbing with soap and water is imperative. The yellow fluid within sulfur mustard blisters may cause secondary cutaneous injury upon rupture.^4^

Management is largely symptomatic treatment and supportive care. Historical treatments such as topical bicarbonate solution and chlorinated soda have been shown to be of no added benefit over soap and water.^5^ Cutaneous burns are managed in a similar fashion to thermal burns with debridement, antibiotic ointment, collagen-laminated nylon dressings and fluid resuscitation as needed. Ocular injuries can be treated with irrigation, antibiotics if secondarily infected, and analgesia. Respiratory symptoms are treated with supplemental oxygen, prophylactic antibiotics, and if needed, mechanical ventilation. Assessment of complete blood count and liver function tests is prudent in the exposed individual due to the potential for serious systemic effects.

Most patients with low concentration cutaneous exposure will survive the injuries, but may go on to have extensive scarring, as the median lethal dose (LD_50_) for cutaneous exposure is 100mg/kg.^4^ Sequelae include hypopigmented areas and scars that can be disfiguring depending on location and extent of injury. Long-term sequelae include development of malignancies later in life, particularly in long-term, chronic low-concentration respiratory exposures (such as in Japanese sulfur-mustard factory workers).^10^,^11^,^12^

The patient exposed to sulfur mustard in our case is projected to have a full recovery, with possible scarring and hypopigmented patches of skin of the right arm, but he will most likely have better cosmesis than victims of decades past due to optimized supportive care and skin grafting. He may experience persistent neuropathic pain, which has been described as a sequela of cutaneous exposure, due to cutaneous afferent nerve damage.^4^ The metabolite of sulfur mustard, thiodiglycol, might have been detectable in his urine for two weeks after exposure if tested, as has been observed in patients with significant cutaneous exposures.^4^

## CONCLUSION

Emergency physicians should be aware of crucial aspects of sulfur mustard exposures, given the potential for exposure at stockpile sites, from inadvertent dredging from the sea floor, or from its potential use as an agent of chemical warfare. Clues for sulfur mustard injury include a reported scent of onions or garlic at the exposure site and the delayed development of bullous lesions containing yellow fluid. Worsening cutaneous injury, pulmonary symptoms or systemic toxicity may be delayed up to 48 hours. Decontamination with soap and water is imperative to prevent further injury to the patient or the healthcare providers. Debridement of cutaneous bullae may cause further cutaneous injury from exposure to blister fluid. Prophylactic antibiotics may be administered in patients presenting with pulmonary symptoms, but care overall remains supportive. Death from sulfur mustard exposure is rare and usually related to large initial exposures or secondary infection developing days to weeks after exposure. After diagnosis, it is imperative to inform local authorities to prevent further exposures.

## Figures and Tables

**Image:**
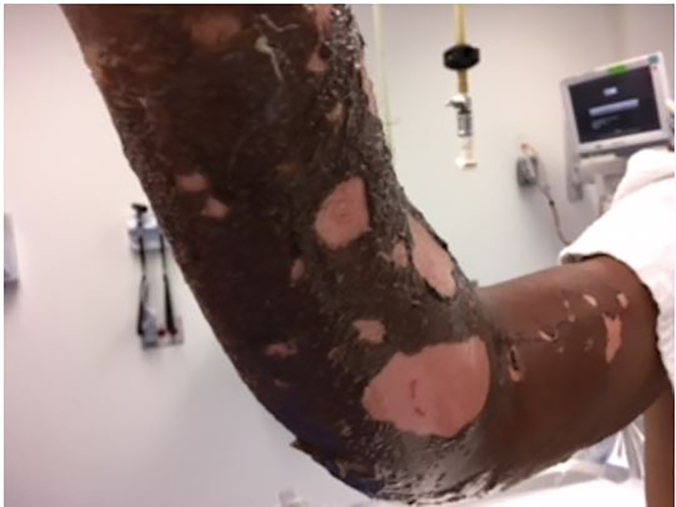
Sloughing bullae of cutaneous sulfur mustard exposure.
